# Development of Event Segmentation in Language and Cognition: Evidence From Dwell Times and Eye Movements

**DOI:** 10.1111/cogs.70212

**Published:** 2026-04-26

**Authors:** Bilge Tınaz, Ercenur Ünal

**Affiliations:** ^1^ Department of Psychology Özyeğin University; ^2^ Multimodal Language Department Max Planck Institute for Psycholinguistics

**Keywords:** Event segmentation, Dwell time, Eye‐tracking, Event Cognition, Motion events

## Abstract

To navigate in and communicate about the continuous world we experience, our minds segment this experience into discrete event units. Yet, languages differ in how they package core aspects of events into linguistic units. Here, we ask how event units in language and cognition relate to each other, and how this relation might change during language acquisition. To do so, we focus on motion events and compare child and adult speakers of Turkish—a verb‐framed language encoding motion events in multiple linguistic units with distinct units for each path segment. In a linguistic task, there were systematic differences in the number of linguistic units used for expressing motion paths when describing events with versus without direction changes in adults and to a lesser extent in 5‐year‐olds but not in 4‐year‐olds. In a non‐linguistic eye‐tracked dwell‐time task, both children and adults had similar visual attention profiles for events with and without direction changes. These findings indicate that although linguistic event units become increasingly language‐specific with age, cognitive event units remain stable and independent of linguistic encoding. These findings show that people flexibly shift between different levels of granularity when segmenting events in language and cognition. Further, this flexibility seems to emerge in children as young as 4 to 5 years old.

## 1 Introduction

The world around us consists of a continuous stream of experience. To make sense of and communicate about it, our minds organize this continuous stream into discrete units known as *events*. This ability emerges early in life, as young children often communicate about the events they experience and observe (Baldwin, Baird, Saylor, & Clark, 2001; Bowerman & Choi, 2001; Pinker, 1989; Saylor, Baldwin, Baird, & LaBounty, 2007; Tomasello & Merriman, 2014). Events can be segmented in different levels of granularity, creating a hierarchical organization, with coarse‐level units subsuming finer‐level units (Zacks, Tversky, & Iyer, 2001). For example, one might construe a trip to a coffee shop as one large event unit or as a series of finer units, including walking into the coffee shop, approaching the counter, placing an order, finding a seat, drinking the coffee, leaving the coffee shop. Similarly, people communicate about events in different levels of granularity. When describing the trip to the coffee shop, one might choose to mention several finer units or describe the entire experience as a single unit. Importantly, these choices are constrained by the language the speaker uses to describe the events, as languages vary in how they package different aspects of events into single units in language. How do event units in language and event units in cognition connect to each other? And how does this relation change developmentally during language acquisition?

### 1.1 Event segmentation in cognition

According to Event Segmentation Theory, our minds readily segment continuous experiences into meaningful event units, which serve as foundations for remembering our experiences, predicting others’ actions, and producing and understanding language (Zacks, Speer, Swallow, Braver, & Reynolds, 2007; see also Radvansky & Zacks, 2017). Working memory representations of events, known as event models, contain information about the core features of an event including its participants and spatiotemporal framework. When core features of the event are stable, the event model can make accurate predictions about upcoming happenings. However, when the core features of the event change, the model cannot accurately predict what will happen next, and the event model has to be updated. This update is perceived as an event boundary. Event boundaries can arise from changes in features such as the people or objects in the event (Zacks, Speer, & Reynolds, 2009), intentionality (Baldwin et al., 2001; Saylor et al., 2007), goal‐related structure (Kosie & Baldwin, 2021), causality (Cohen & Oakes, 1993), and spatial characteristics such as movement of characters (Magliano, Miller, & Zwaan, 2001), direction (Zacks, 2004), and trajectories of motion (Shipley & Macguire, 2008). Others have proposed that event boundaries can be also formed from anticipating changes to the core features of the event (Wang, Ongchoco, & Scholl, 2023; see also Baldwin & Kosie, 2021; Shin & DuBrow, 2021).

Event segmentation is typically measured by eliciting explicit judgments of event boundaries. In a classical paradigm by Newtson (1973), participants are instructed to watch a movie showing an actor performing several activities and to indicate each new unit with a button press. Studies using this paradigm have shown that people consistently identify the breakpoints in the event in a hierarchical manner, at fine‐grained and coarse‐grained levels (Sargent et al., 2013; Sasmita & Swallow, 2023; Zacks et al., 2001). Furthermore, the movie frames that correspond to the breakpoints are remembered better than the frames that correspond to within‐unit moments (Newtson & Engquist, 1976). However, one caveat of this method is that it is an explicit measure of event segmentation as it requires participants to make a behavioral response reflecting their judgment as well as substantial verbal instruction and explanation about events and what “fine” and “coarse” event units might correspond to.

This caveat is addressed by the Dwell Time paradigm by Hard, Recchia, and Tversky (2011). Participants are presented with a series of still images sampled from videos of continuous actions at regular intervals and asked to advance through this slideshow at their own pace by pressing a button. The time between the button presses is recorded as an index of how long they view each slide. Studies using this method have shown that slides that are judged to be event boundaries are viewed for a longer time, compared to the slides that fall between event boundaries (Hard et al., 2011; Kosie & Baldwin, 2021; Meyer, Baldwin, & Sage, 2011; Sage & Baldwin, 2014; Zheng, Zacks, & Markson, 2020). This increase in viewing time is attributed to the additional demand for attention at event boundaries that is required for consolidating event representations in memory.

Due to its relatively more implicit nature, the Dwell Time paradigm has been deemed suitable for use with diverse populations, including children as young as 2.5 years old (Kosie & Baldwin, 2021; Meyer et al., 2011; Zheng et al., 2020). Using this method, Kosie and Baldwin (2021) showed that 2.5‐ to 4.5‐year‐olds had increased viewing times for slides that were also explicitly judged as event boundaries and prioritized goal‐related structure over motion patterns, indicating an early sensitivity to the internal structure of the event. Consistent with this, other studies have shown that children, like adults, allocate more attention to event boundaries than within‐event moments and can identify boundaries at both a fine‐grained and a coarse‐grained level, when prompted (Meyer et al., 2011; Zheng et al., 2020).

### 1.2 Event segmentation in language

According to many accounts of speech production, the main unit of processing in language corresponds to the information that can be encoded in a clause or a verb phrase in a given language (Bock, 1982; Levelt, 1989; Norcliffe & Konopka, 2015). Yet, there is considerable cross‐linguistic diversity in the clausal‐level packaging of core event components. For example, an event can be expressed in the same level of detail with a single clause in one language but might require multiple clauses in another. It has been proposed that this variation might reflect different conceptualizations of the same event with single‐clause descriptions conceptualizing the event as one unit and multi‐clause descriptions implying that it consists of several smaller units (Croft, 1991; Levin & Rapoaport Hovav, 1999).

A well‐attested case of this variability is motion events (Bohnemeyer et al., 2007; Talmy, 2000). Motion events consist of an entity changing location with respect to a landmark or along a trajectory (path) and in a certain manner. Talmy's (2000) typology classifies languages into two categories based on how they encode path of motion. In satellite‐framed languages (e.g., English, German, Dutch), manner of motion is typically expressed in the main verb, while path is expressed through satellites of the verb (e.g., particles or adpositions). Since manner verbs can be freely combined with different path segments, satellite‐framed languages can tightly package manner and path into a verb phrase, even if there are multiple path segments (see Example 1 from English). By contrast, in verb‐framed languages (e.g., Turkish, Greek, French) path of motion is typically expressed in the main verb, while manner of motion is optionally expressed through adverbs, subordinate verbs or adpositions. As a result, each path segment or change in the direction of motion is expressed in a new verb phrase (see Example 2 from Turkish).

(1)
The  ball  rolled    down     into     the house
        verb    preposition  preposition
        manner   path     path

(2)
Top  yuvarlan‐arak  in‐di     ve   ev‐e     gir‐di
 ball  roll‐conn    descend‐pst  and  house‐dat  enter‐pst
     sub. verb     verb              verb
     manner      path              path
     “The ball rolling (and) descended and entered the house.”

A slightly different typological classification by Bohnemeyer et al. (2007) distinguishes further between different types of path or trajectory changes. One type of change corresponds to events of departure, arrival, and passing, which involve a change to the landmark (or ground) object that marks the trajectory of motion. Type‐I languages—similar to satellite‐framed languages—can package these three subevents into a single clause. By contrast, Type‐II languages—similar to verb‐framed languages—can package arrival and departure together but often separate passing. Finally, Type‐III languages exclusively use separate clauses for each path subevents and lack constructions that can integrate multiple path subevents into a single clause. Another type of change concerns the changes in direction in which path of motion consists of two or more non‐collinear direction vectors (Bohnemeyer, 2003). Such direction changes have further subtypes. For some direction changes consisting of two or more non‐collinear direction vectors, the description of the path is segmented into multiple units in any language (e.g., *The woman walked down the street and turned into the coffee shop*). Others show more cross‐linguistic variability (Carroll, Lambert, Weimar, Flecken, & Stutterheim, 2012; Gerwien & von Stutterheim, 2018). For such direction changes, in verb‐framed languages, the description of the path is typically segmented into multiple units with one unit per each direction vector (as in Example 2 above). For satellite‐framed languages, it is also possible for the direction change to not be explicitly expressed and derived by implicature, resulting in the description of the path integrated into a single unit (as in Example 1 above). Although this classification offers a more fine‐grained distinction on the type of path changes, for present purposes, it leads to the same broad prediction on how motion paths are segmented into linguistic units as we focus on the latter subcategory that is subject to cross‐linguistic variability.

Several cross‐linguistic studies with typologically different languages have demonstrated that adults indeed adhere to their language's lexicalization patterns when describing motion events (e.g., Bohnemeyer et al., 2007; Papafragou, Massey, & Gleitman, 2002, 2006; Slobin, 1996; Ünal, Manhardt, & Özyürek, 2022; see also studies in Bylund & Athanasopoulos, 2015; Ibarretxe‐Antuñano, 2017; Ünal, Mamus, & Özyürek, 2024). However, children's motion event descriptions are characterized by both language‐general and language‐specific patterns. Around age 3, children begin showing sensitivity to the typology of their native language (Allen et al., 2007; Bunger, Trueswell, & Papafragou, 2012; Hickmann, Taranne, & Bonnet, 2009; Maguire et al., 2010; Özçalışkan & Slobin, 1999; Özyürek et al., 2008). For instance, English‐speaking 3‐year‐olds often express path and manner in a single clause, whereas Turkish‐speaking children often express path and manner in separate clauses (Allen et al., 2007; Özyürek et al., 2008). However, these patterns gradually emerge, and young learners of typologically different languages also show similarities. For example, 3‐year‐olds acquiring English, Japanese, and Turkish initially produce simple clauses that encode single event components, with more complex clausal constructions emerging later in development (Allen et al., 2007). This suggests constraints on early uses of clausal‐level packaging that generalize across languages. Supporting this, Bunger et al. (2012) found that 4‐year‐old English speakers were more likely than adults to omit event components (especially path of motion), even though they attended to both event components in similar temporal profiles as adults, suggesting limitations on young children's language production system.

### 1.3 Linguistic influences on event segmentation

Could these language‐specific patterns influence how people segment events in non‐linguistic cognition? This question connects to a broader discussion on the relation between language and cognition (for reviews, see Wolff & Holmes, 2011; Ünal & Papafragou, 2016). According to one position, known as linguistic relativity, lexical, semantic or syntactic distinctions in language create stable differences in how users of different languages reason about conceptual categories, even when they are not using language (Levinson, 2003; Majid, Bowerman, Kita, Haun, & Levinson, 2004; see Whorf, 1956, for the initial statement of this position). On an alternative view, language builds on conceptual categories that are shared across people cross‐linguistically to a large extent (Gleitman & Papafragou, 2016; Landau, Dessalegn, & Goldberg, 2010). This view also acknowledges that language may influence non‐linguistic cognition while translating thoughts into language but not in the absence of explicit or implicit language use.

Until recently, how people segment events into units in language and cognition have been studied independently, by different groups of scholars, and using different methodologies. As a result, these competing views on the relation between language and cognition could not be tested in the domain of event segmentation. This gap was addressed in a cross‐linguistic study on event segmentation comparing speakers of Dutch and Avatime (Defina, 2016). Unlike Dutch, Avatime, a Kwa language spoken in Ghana, is known for its pervasive use of serial verb constructions. In such constructions, two or more verbs appear consecutively and form a compound structure that refers to a single event. In order to test whether Avatime speakers would segment events more coarsely than Dutch speakers, speakers of both languages completed a Dwell Time task in which they completed self‐paced slideshows depicting familiar and unfamiliar events. There were no cross‐linguistic differences in dwell times; however, both groups segmented the events they were familiar with differently than the events they were unfamiliar with. In another study, Avatime speakers were primed with either serial verb constructions or coordinate clauses before the Dwell Time task, which resulted in differences in non‐linguistic event segmentation behavior. Overall, these findings suggest that under conditions of priming, language‐specific constructions influence non‐linguistic segmentation, though such influences do not seem to be stable across contexts. However, one limitation of this study was that the description task for establishing cross‐linguistic differences in linguistic expression and the Dwell Time task relied on different stimuli and events. A stronger test of linguistic influences on non‐linguistic event segmentation would involve comparing how people segment the very same events in language and cognition.

A recent study by Gerwien and von Stutterheim (2018) addressed this limitation by comparing how adult native speakers of French (a verb‐framed language) and German (a satellite‐framed language) form linguistic and non‐linguistic event units for the very same events. In the linguistic task, French speakers were more likely than German speakers to use more than one verb phrase to describe the motion events that had a change in direction. In the non‐linguistic task, another group of French and German speakers completed the Newtson (1973) task. French speakers were more likely than German speakers to indicate that there was an additional event boundary for events that involved a change in direction. These cross‐linguistic differences were taken as evidence for the presence of strong language‐driven influences on cognitive event unit formation.

Even though these findings are suggestive of a parallel between event units in language and cognition, some aspects of the study challenge this interpretation. First, in the linguistic task, the descriptions were only coded in terms of whether events were described using more than one verb phrase. However, whether these verb phrases expressed path and/or manner of motion were not taken into account. This is important because, although verb‐framed languages typically encode manner outside of the main verb and there are fewer manner verbs available, it is still possible to express manner of motion in verbs. Thus, it might be possible to produce a description consisting of more than one verb phrase without using a new phrase to refer to a different path segment but simply by expressing manner and path in separate verb phrases (e.g., rolled and entered). This is noteworthy because such distinctions encode two motion components occurring simultaneously, which somewhat diverges from how event‐segmentation theories typically characterize events (Radvansky & Zacks, 2017). On this view, events units are discrete and contiguous, forming a sequence in which one event ends, and the next one begins. This does not imply that events must be contiguous non‐overlapping temporal units but simply highlights that how temporally overlapping changes are segmented is different from the typological and linguistic distinctions examined in earlier work.

Further, it is possible that participants in the non‐linguistic task may have been implicitly verbalizing even though they were not explicitly required to respond verbally. This is especially likely for the Newtson task in which participants provided boundary judgments by interpreting verbal instructions (i.e., to press a button “when [they] perceive a change/whenever something new happens”). Due to its explicit nature, this task might have encouraged participants to make a communicative inference about what is intended to be construed as “a change” or “something new” and to consult the way their language linguistically segments motion paths (cf., Gleitman & Papafragou, 2016; Li, Abarbanell, Gleitman, & Papafragou, 2011). Thus, participants may have been engaging in linguistic encoding—at least to some extent—during non‐linguistic event segmentation, even if the response was non‐verbal (i.e., button press). In fact, previous work on other aspects of cognition has shown that cross‐linguistic differences diminish or disappear when people are prevented from implicitly using language while performing a non‐linguistic task (e.g., Trueswell & Papafragou, 2010; Winawer et al., 2007) or when presented with other non‐linguistic cues (Li et al., 2011). Although the tasks used in these studies are very different from the tasks used for measuring event segmentation, the patterns observed in other domains raise the possibility that the cross‐linguistic differences in event segmentation reported earlier might reflect thinking with language rather than only non‐linguistic cognition. Thus, an open question is whether language‐specific event units would be reflected in how events are segmented non‐linguistically when the explicit (including linguistic) demands of the task are minimal.

A second question arising from the present findings is whether language‐specific event units would influence non‐linguistic event segmentation in children during language acquisition. Previous developmental work has shown that young children's event descriptions are characterized by both language‐general and language‐specific patterns, though they gradually become more language‐specific as they get older (Allen et al., 2007; Hickmann et al., 2009; Maguire et al., 2010; Özçalışkan & Slobin, 1999; Özyürek et al., 2008). A separate line of work on event segmentation in children has shown that children can segment various types of events in different levels of granularity (Kosie & Baldwin, 2021; Meyer et al., 2011; Zheng et al., 2020). The question is how these two abilities make contact with each other. If children learn a language that encodes motion paths in distinct units, do they also segment events into finer‐level units based on the direction changes in the event? Investigating children's linguistic and non‐linguistic event segmentation can also provide insights that research with adults cannot, as adults’ mature cognitive abilities may allow them to flexibly adapt different levels of granularity in linguistic vs. non‐linguistic event segmentation. However, linguistic categories may exert their influence more strongly during acquisition (cf., Bowerman & Choi, 2001; Bowerman & Levinson, 2001), increasing children's cognitive sensitivity to the distinctions encoded in their language while these distinctions are acquired.

### 1.4 The current study

Our goal here is to further investigate the scope of linguistic influences on non‐linguistic event segmentation. We investigate whether clausal‐level packaging of event units in language might influence event segmentation in cognition even under minimal explicit and linguistic demands. We also ask if such influences might change throughout language development, as children become more proficient in the typological patterns of their native language. To do so, we focus on Turkish—a verb‐framed language that expresses motion events in multiple clauses with separate verb phrases for each path segment. We used motion events that involve a change in direction and hence consist of multiple path segments. As a control, we also included motion events that do not involve a change in direction. We used the same stimuli across a linguistic description task and a non‐linguistic Dwell Time task (Hard et al., 2011) completed by the same participants. We conducted developmental comparisons between adults and children from two age groups: 4‐year‐olds and 5‐year‐olds. For children, we focused on these age groups because previous work showed that 4‐year‐olds still frequently omit event components from their descriptions and often produce single‐clause descriptions (Allen et al., 2007; Bunger et al., 2012). These within‐language comparisons between tasks and age groups allowed us to test whether and under what circumstances linguistic and non‐linguistic event units were similar to as well as different from each other.

We begin with a detailed examination of children's and adults’ linguistic descriptions. As described in the previous section, in verb‐framed languages motion events can be described with multiple clauses not only by encoding each path segment with a different verb phrase but also by expressing path and manner in separate clauses. To address this limitation, we inspect the semantic content of the descriptions and check whether the descriptions with multiple verb phrases indeed encode a change in direction. Given that Turkish typically expresses each path segment with a separate verb phrase, we expected participants to produce descriptions that consist of multiple verb phrases more frequently for events that involve a direction change than those that do not involve a direction change. Further, this difference should persist when the semantic content of the descriptions is considered, such that events involving a direction change should be more likely to be described with more than one path verb. However, since language‐specific structures may not be fully acquired by age 4 or even 5, we expect the use of descriptions consisting of multiple verb phrases to increase with age.

In the non‐linguistic task, participants completed a Dwell Time task (Hard et al., 2011). In this task, participants were simply instructed to advance through a slideshow at their own pace—a procedure that is less likely to encourage communicative inferences that would encourage participants to rely on linguistic encoding and therefore can be considered as a more implicit measure of event segmentation than explicit boundary judgments. Assuming that the predicted patterns surface in linguistic descriptions of adults and children, we can distinguish between four broad possibilities for the non‐linguistic task. If linguistic event units influence how people segment events non‐linguistically, participants should segment events that involve a direction change and those that do not differently. One possibility is that this effect could be stronger in adults than in children, and possibly in older children than in younger children. This would indicate that the long‐term experience with the linguistic encoding of motion paths into finer units increases sensitivity to motion paths in non‐linguistic cognition in adults, but that this influence is not yet present in children. An additional possibility is that this effect might only emerge in children. This would suggest that learning to encode motion paths into finer units in language increases sensitivity to motion paths in non‐linguistic cognition during acquisition, but this sensitivity does not persist in proficient users (adults).Alternatively, both children and adults may segment events that involve a direction change differently than those that do not, which would indicate that cognitive ability to segment such events develops prior to full mastery of the corresponding linguistic distinctions and before the age range tested in this study. Finally, the number of event units in the segmentation task may not necessarily parallel the number of event units in language. If so, dwell time patterns are expected to be similar across event types and age groups, even if they are described differently. This last possibility would indicate that clausal‐level packaging of event units in language does not influence event segmentation in cognition.

## 2 Method

### 2.1 Participants

Data were collected from 94 native speakers of Turkish across three age groups: adults (*n* = 31, 21 females, *M*_age_ = 21.96, *SD*_age_ = 2.41, range = 18.87–27.84), 4‐year‐olds (*n* = 31, 24 females, *M*_age_ = 4.44, *SD*_age_ = 0.39, range = 3.69–4.99), and 5‐year‐olds (*n* = 32, 21 females, *M*_age_ = 5.58, *SD*_age_ = 0.32, range = 5.17–6.29). Children were recruited from private and public kindergartens in Istanbul, Turkey. The medium of education in these kindergartens was Turkish. Adults were undergraduate students at Özyeğin University in Istanbul and received course credits for their participation. Data from 12 additional participants (nine children, three adults) were excluded for the following reasons: not being a native speaker of Turkish (one adult), not following the instructions (seven children, two adults), or being older than 6 years (two children).

### 2.2 Materials

**Fig. 1:**
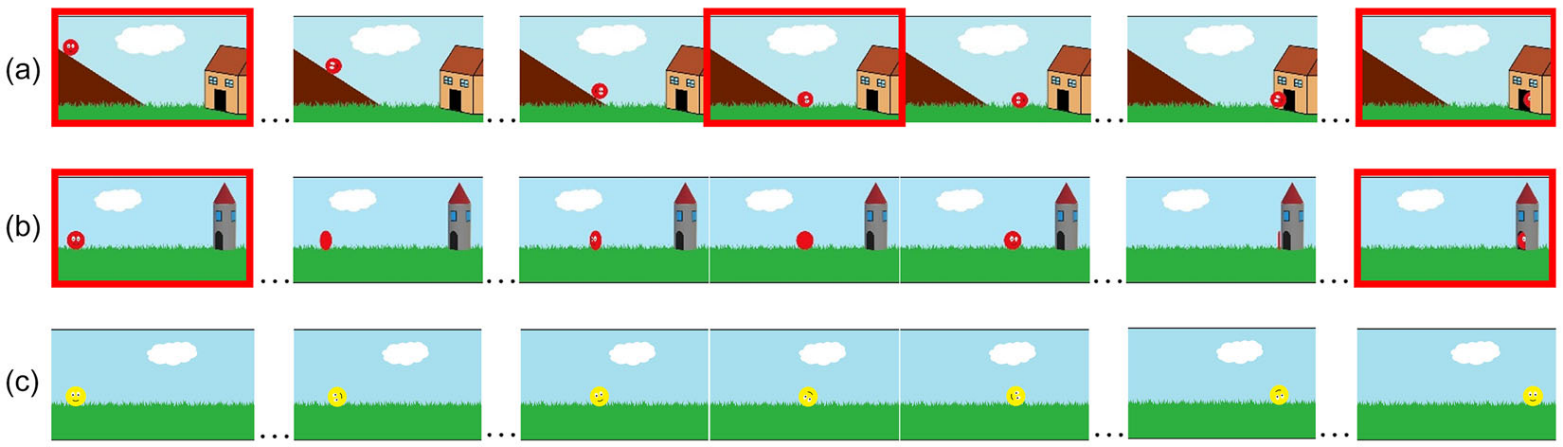
Examples of boundary (outlined in red) and non‐boundary slides for events involving (a) path‐change, (b) no path‐change, and (c) filler events.

Stimuli consisted of nine videos of motion events. Target stimuli consisted of videos of six motion events depicting objects changing location in a specific manner and along a trajectory with respect to a landmark object. There were two types of target motion events: events that involved a path change (e.g., a ball rolled down a hill and moved along the lateral axis to enter into a house; Fig. 1a; three events in total) and events that did not involve a path change (e.g., a ball spun along the lateral axis and entered in a tower; Fig. 1b; three events in total). Three additional videos of motion events served as fillers. Filler events were visually similar to the target events but crucially did not involve any path change and any landmark that would mark the trajectory of motion (e.g., a triangle jumping to the left; Fig. 1c). Filler events were included to increase the overall number and variability of the stimuli and the data from these trials were not included in the analysis. Stimuli are available at https://osf.io/ugmyq.

Stimuli were created in Adobe Premiere Pro CC 2015. Each video was 10 s long. Videos featured a sky‐blue background with a cloud and green grass on the ground. Each video incorporated a landmark object, combined with a moving figure to establish distinct motion paths. For *into* paths, landmarks were positioned near the motion's endpoint. For *past* paths, landmarks were placed toward the motion's end in a way that allowed the moving object to pass them. Paths involving *upward* or *downward* motion were represented using a hill or stairs.

For path‐change events, the scene was designed to include a brief period of unpredictability. At the base of each included surface, the ground surface extended beyond the incline in both directions. For example, in Fig.1a, the grass continues past the beginning of the hill. This meant that when the moving object reached the base of hill, multiple continuations were plausible (e.g., rolling into the house vs. rolling in front of the hill, or continuing straight vs. moving up the incline), and the change in direction was not visually predetermined. This feature introduced a short window of unpredictability prior to the direction change and ensured that participants could not predict a single upcoming trajectory solely from the static scene.

Each of the three figure objects appeared once within each stimuli type (path‐change, no path‐change, and filler events). There were no other constraints for combining the figure objects with individual manners or paths of motion. In order to ensure that the changes in dwell times would be triggered by changes in the direction of motion (cf., Meyer et al., 2011; Zheng et al., 2020), the camera angle and other low‐level perceptual features of the scene, except for the movement of the figure, remained stable throughout the entire video.

For the non‐linguistic task, we created a slideshow by sampling screenshots from the videos at 1‐s intervals. Thus, each 10‐s video was converted into a slideshow consisting of 11 images (see Fig. 1). For path‐change events, the slide depicting the change in direction was set to be in the middle, that is, on the sixth slide, for each slideshow.

To determine the appropriate stimulus length, we reviewed dwell‐time and self‐paced viewing paradigms across adult and child populations. Although studies with adults have typically used long slideshows (range = 108–298 slides; e.g., Hard et al., 2011; Kosie & Baldwin, 2019b; Sage & Baldwin, 2014), developmental work shows substantial variability in slideshow length, and typically using fewer images per slideshow (range = 20–95 slides; e.g., Kosie & Baldwin, 2021; Meyer et al., 2011; Sage & Baldwin, 2015; Zheng et al., 2020). Because our events involved a single continuous motion path with one potential mid‐event boundary, we opted for a shorter slideshow of 11 images.

#### 2.2.1 Norming study

Prior to the main experiments, event boundaries that corresponded to path changes were independently verified in a norming study. Data were collected from 30 adult native speakers of Turkish who did not participate in the main experiment. Following Kosie and Baldwin (2021), participants were provided with definitions of events and event boundaries, along with verbal examples. They were instructed that an event could be viewed as a single event or as smaller sub‐events, each considered a separate event. Participants were shown the complete sequence of slides in order and asked whether each slide depicted a boundary (coded as 1) or not (coded as 0). For each slide, we calculated the proportion of responses that indicated the presence of a boundary. Mean boundary judgments for the path‐change slide (Slide 6) and the slide leading up to the path change (Slide 5) were significantly higher for events that involved a path change (*M* = 0.46) as opposed to the events that did not involve a path change (*M* = 0.30; *χ^2^* (1) = 9.238, *p* = .002).

### 2.3 Procedure

Children were tested individually in a quiet room at the kindergartens. Adults were tested individually in a lab at their university campus. Participants were seated approximately 60 cm away from a DELL Precision M4800 laptop with the SMI RED 250 eye‐tracker (SensoMotoric Instruments) mounted underneath the screen. Eye gaze was sampled (binocular) at a rate of 250 Hz. Screen resolution was 1920 × 1080. The size of the stimuli were 1920 × 1080 pixels. The stimuli were presented via NBS Presentation software (Version 23.1, Neurobehavioral Systems, Inc., www.neurobs.com).

All participants received both the linguistic and non‐linguistic Dwell Time task and in a fixed order: They performed the non‐linguistic task first and the linguistic task second. This was done to prevent transfer from a task that involves using language to one that does not.

#### 2.3.1 Linguistic task

Participants watched nine video clips of motion events displayed on the computer screen. In each trial, participants first saw a fixation cross for 1000 ms, followed by the video of an event that lasted 10 s. Finally, a gray screen appeared, during which participants described what happened in the video to a confederate addressee who was seated across them and could not see the computer display. Descriptions were recorded (video and audio for adults, audio only for children) for later coding. For adults, the experimenter handed the mouse to the confederate after the practice trials, and the addressee clicked on the mouse once the participant finished describing to initiate the next trial. For children, the experimenter sat beside them throughout the experiment and initiated the next trial with a mouse click. This was done to prevent children from advancing to the next trial before finishing their descriptions. There were no other differences in the procedure for children and adults. Individual items were presented in a single randomized order, with half of the participants receiving the items in the original order and the remaining half receiving the items in the reversed order. Prior to the main experiment, participants completed two practice trials, followed by optional feedback and the opportunity to ask questions.

#### 2.3.2 Non‐linguistic Dwell Time task

Participants were instructed that they would see pictures on the screen and that they could advance through the images at their own pace by pressing the spacebar on the keyboard. They were asked to keep their heads as still as possible and their hands on the table. Each participant saw a total of nine slideshows. Individual slideshows were presented in a single randomized order with half of the participants receiving the original order and the other half receiving the reversed order. The randomized order of items in the non‐linguistic task was different than the order in the linguistic task.

Prior to the main task, participants completed a practice slideshow depicting screenshots from a video of a penguin throwing a snowball, familiarizing them with the process of pressing the spacebar to watch an event unfold. After the practice trials, a 5‐point calibration and validation procedure were completed. Following an opportunity to ask questions, the main task began.

For adults, the experimenter sat in a chair behind the participants. For children, the experimenter sat beside them to ensure their attention remained focused, they did not look elsewhere, or randomly click through the slideshow. Furthermore, a pilot study revealed that children had difficulty reaching the spacebar without moving too much. Hence, they were asked to navigate through the slideshows with a mouse click.^1^ There were no other differences in the procedure for children and adults.

Dwell time was recorded with two measures: the latency between button presses indicating the amount of time a slide was visible on the screen and the total duration of fixations recorded by the eye‐tracker indicating the amount of time a slide was attended to. The latency between button presses has been used in previous work as an implicit measure of the perception of event boundaries, with longer latencies indicating longer viewing time (Hard et al., 2011; Kosie & Baldwin, 2021; Meyer et al., 2011; Zheng et al., 2020). The total duration of fixations as recorded by the eye‐tracker was added as an additional estimate of the attention allocated to each slide. We included this additional measure because in prior work, the latency between button presses in the self‐paced slideshow has been referred to as “looking time” (Hard et al., 2011), even though no direct measure of looking behavior had been used. It is reasonable to use the amount of time a slide appears visible on the screen as a proxy of looking time or how long each slide is viewed, especially for adults. Children, however, might be more easily distracted than adults, and therefore the amount of time a slide remains visible on the screen may not be identical to the amount of time they spend viewing it. Therefore, as an additional measure, we recorded and analyzed looking behavior using an eye‐tracker. As for the specific eye‐tracking measure, we calculated the sum of the duration of all fixations to a slide (i.e., total fixation durations), which indicates for how long a given slide was viewed.^2^

### 2.4 Coding

Descriptions were transcribed and coded by native Turkish speakers on ELAN (Lausberg & Sloetjes, 2009). We coded the number of linguistic units participants used when describing the event. A linguistic unit was defined as one finite verb that referred to the motion event shown in the video (cf., Gerwien & von Stutterheim, 2018). That is, we included main verbs of sentences or clauses that were tensed and had agreement with the subject (e.g., *the ball descended*) but excluded adverbs. Additionally, we examined the semantic features of the descriptions that consisted of more than one verb phrase. Specifically, we coded whether or not descriptions consisting of more than one verb phrase included at least two different path verbs and hence expressed the change in direction. For instance, Example (2) from Turkish above consists of both multiple verb phrases and multiple path verbs *indi* “descended” and *girdi* “entered”. Example (3) from Turkish also consists of multiple verb phrases, but the first verb *yuvarlandı* “rolled” expresses manner, while the second verb *girdi* “entered” expresses path of motion. Hence, the description in Example (3) does not express the change in the direction of motion. We only coded descriptions similar to Example (2) as including at least two different path verbs.

(3)
Top  yokuş‐tan  aşağı  yuvarlan‐dı  ve   ev‐e     gir‐di
 ball  hill‐abl   down  roll‐pst    and   house‐dat  enter‐pst
             verb              verb
             manner             path
    “The ball rolled down the hill and entered the house.”

### 2.5 Preprocessing of the eye movement data in the Dwell Time task

A message was sent from the Presentation software to the eye‐tracker to indicate the onset and offset of each slide. A rectangular area of interest (AoI) was defined to cover each slide using SMI BeGaze software, to ensure participants’ attention was directed to stimuli. Fixation durations to the AoI were computed by the SMI BeGaze software and aggregated for each slide prior to the analysis.

Data from individual trials with more than 50% trackloss were excluded from the analysis (3.82% of all data, children: 3.03%, adults: 0.75%). None of the participants had to be excluded based on the a priori criterion of having more than 45% trackloss throughout the entire task.

## 3 Results

Data were analyzed with linear mixed effects modeling with crossed random intercepts for Subjects and Items. More complex models that included the maximal random‐effects structure justified by the design of the study (Barr, Levy, Scheepers, & Tily, 2013) failed to converge, and thus random slopes were omitted. The models were fit with the *lme4* package (version 1.1.33; Bates, Machler, Bolker, & Walker, 2015) in R (version 4.2.3; R Core Team, 2023). Figures were produced using ggplot2 package (version 3.4.2, Wickham, 2016). Data and analysis code are available at https://osf.io/ugmyq/.

In order to test whether the number of event units formed in linguistic and non‐linguistic tasks differed across event types and age groups, the fixed effects of these variables were tested with planned comparisons using contrast coding schemes (Schad, Vasishth, Hohenstein, & Kliegl, 2020). The fixed effect of event type was tested with sum‐to‐zero contrasts (path change‐coded as −1/2, no path change‐coded as +1/2). The fixed effect of age was tested using two orthogonal sum‐to‐zero contrasts. The first contrast compared younger children to older children (4‐year‐olds coded as −1/2, 5‐year‐olds coded as +1/2, adults coded as 0). The second contrast compared children to adults (4‐year‐olds coded as −1/3, 5‐year‐olds coded as −1/3, adults coded as +2/3).

### 3.1 Linguistic task

**Fig. 2:**
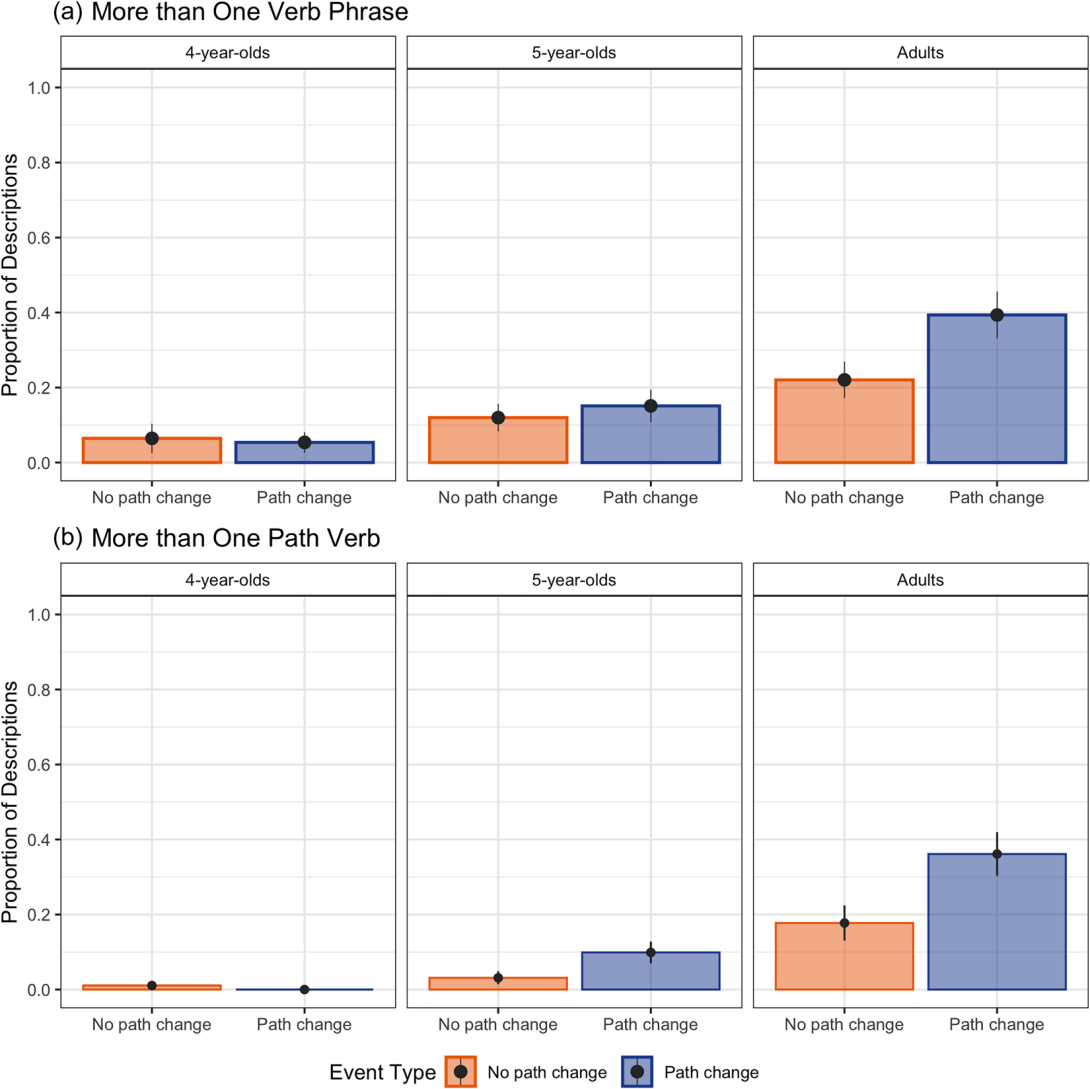
Proportion of descriptions with (a) more than one verb phrase and (b) more than one path verb across event types and age groups. Error bars represent the standard error of participant means.

First, we examined the variation in the number of verb phrases used by participants when describing different types of events (Fig. 2a). The model tested fixed effects of event type and age on the binary dependent variable of using more than one verb phrase (1 = present, 0 = absent) at the trial level. The parameter estimates from the *glmer* model are presented in Table 1. The model revealed that the fixed effect of age was statistically significant for both contrast levels. This indicated that 5‐year‐olds were more likely to describe events with more than one verb phrase compared to 4‐year‐olds. Furthermore, adults were more likely to describe events with more than one verb phrase, compared to children. No other effects or interactions were statistically significant, indicating that these patterns were similar across events that involved a path change and events that did not involve a path change.

**Table 1:** Parameter estimates for the fixed effects from the *glmer* model for using more than one verb phrase

Effect	ß	*SE*	*z*	*p‐*Value
Intercept	−2.499	0.343	−7.288	< .001
Event type _(no path change vs. path change)_	0.496	0.476	1.042	.297
Age _(4‐year‐olds vs. 5‐year‐olds)_	1.237	0.602	2.056	.040
Age _(children vs. adults)_	2.014	0.464	4.340	< .001
Event type _(no path change vs. Path change)_: age _(4‐year‐olds vs. 5‐year‐olds)_	0.050	0.882	0.056	.955
Event type _(No path change vs. Path change)_: age _(children vs. adults)_	0.944	0.589	1.602	.109

Next, we tested if the number of path verbs used in speech differed depending on event type and age (Fig. 2b). Note that all descriptions that consist of more than one path verb also consist of multiple verb phrases. Thus, any differences in more than one path verb use would indicate differences in the number of linguistic units used for encoding path segments. When participants produced descriptions that consisted of more than one verb phrase, 4‐year‐olds used more than one path verb extremely rarely (1% of the descriptions). For that reason, the data from 4‐year‐olds were not included in the model. For completeness, these data are included in Fig. 2b. The remaining data were analyzed with a *glmer* model testing the fixed effects of event type (path change, no path change) and age (5‐year‐olds, adults) on the binary dependent variable more than one path verb (1 = present, 0 absent) at the trial level. The fixed effects of event type and age were tested with sum‐to‐zero contrasts (−1/2, +1/2). Parameter estimates from the *glmer* model are presented in Table 2. The model revealed a significant effect of age: adults were more likely to use more than one path verb compared to 5‐year‐olds. The fixed effect of event type was also statistically significant: Participants were more likely to use more than one path verb in descriptions of path‐change events, compared to no path‐change events. No other effects or interactions were statistically significant.

**Table 2:** Parameter estimates for the fixed effects from the *glmer* model for using more than one path verb

Effect	ß	*SE*	*z*	*p‐*Value
Intercept	−2.340	0.328	−7.139	< .001
Event type _(no path change vs. path change)_	1.238	0.456	2.716	.007
Age _(5‐year‐olds vs. adults)_	2.013	0.529	3.802	< .001
Event type _(no path change vs. path change)_: age _(5‐year‐olds vs. adults)_	−0.012	0.806	−0.015	.988

### 3.2 Non‐linguistic Dwell Time task

First, we tested the correlation between the two measures of dwell time: the latency of the button presses between each slide and the total duration of fixations to a slide. There was indeed a strong positive correlation overall between these two measures (*r* = 0.87), for both adults (*r* = 0.88) and children (*r* = 0.86), indicating that they provide highly similar but not identical estimates of dwell times. We began by analyzing the latency between the button presses as an index of dwell time or the amount of time a slide was visible on the screen as used in previous work (Hard et al., 2011; Kosie & Baldwin, 2021; Meyer et al., 2011; Zheng et al., 2020). We then analyze the total duration of fixations or the time participants spent viewing each slide as an additional measure of dwell time.

#### 3.2.1 Latency between button presses

Analyses were performed on log‐transformed button‐press dwell times and excluding trials that had dwell times 3 standard deviations above or below the mean (0.72% of all data, children: 0.57%, adults: 0.15%; Kosie & Baldwin, 2019a, 2019b, 2021). Data were inspected to see if participants speeded up over time (i.e., showed a power curve). This was not the case, and thus fitting a power function and residualizing was not necessary (see Kosie & Baldwin, 2021, for a similar approach).

**Fig. 3:**
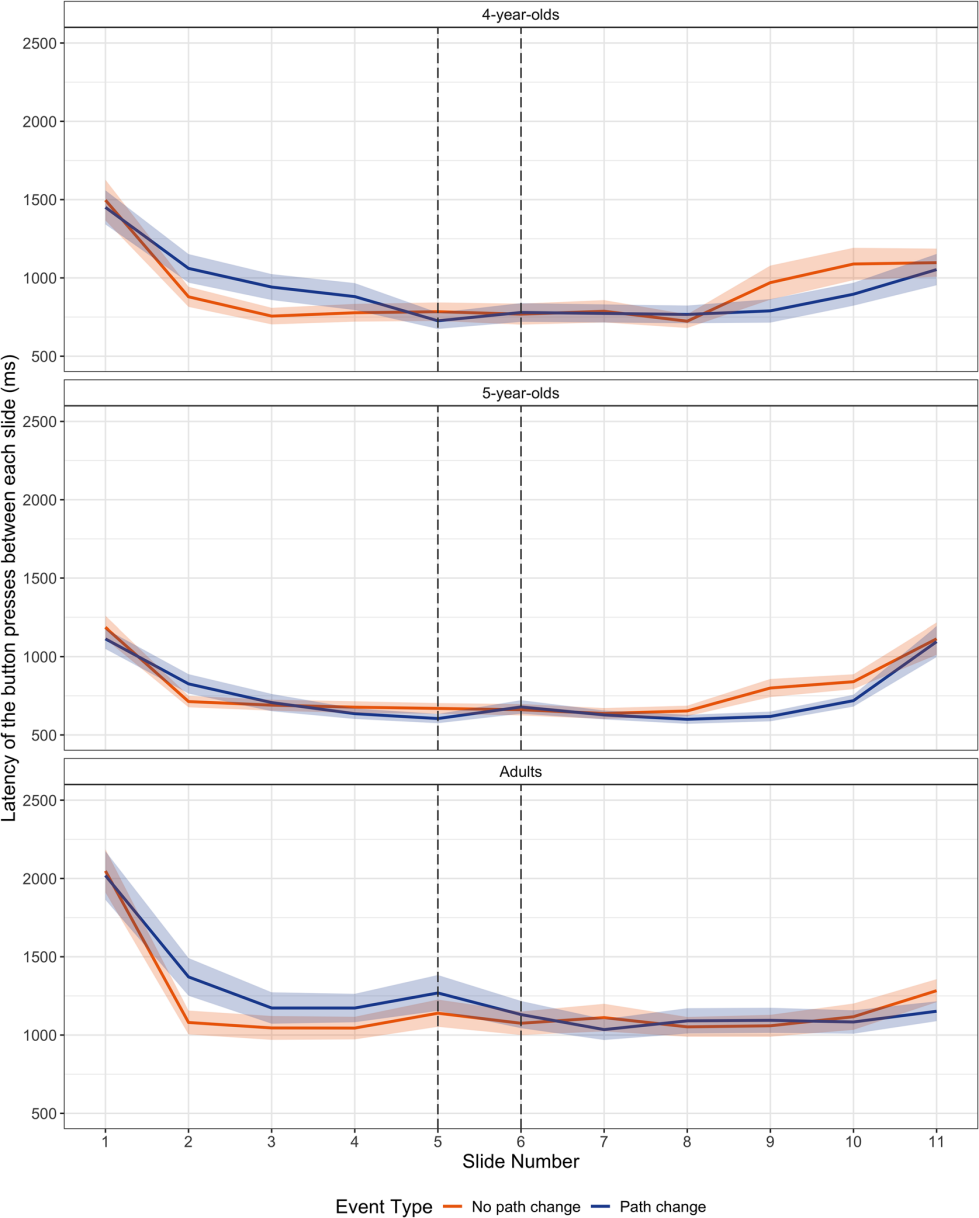
Latency of the button presses between each slide across event types and age groups. Shaded areas represent the standard error of participant means.

We tested whether the events that involved a change in direction were perceived as having an additional event boundary as indicated by increases in the latency of the button presses for the slides depicting a path change and the slide leading up to the path change (Slides 5 and 6) but not for temporally similar slides for no path‐change events. The model tested the fixed effects of event type on the latency between the button presses to the two middle slides as the dependent measure (Fig. 3). Parameter estimates from the model are presented in Table 3. The model revealed a significant fixed effect of age at the second contrast level, indicating that adults overall had longer latencies between button presses and thus dwelled on the slides for longer than children did. Importantly, there were no effects or interactions involving event type. This indicated that button‐press dwell times to slides that depicted a path change were similar to temporally similar slides that did not depict a path change.

**Table 3:** Parameter estimates for the fixed effects from the *lmer* model for the latency of the button presses

Effect	ß	*SE*	*df*	*t*	*p‐*Value
Intercept	2.839	0.029	17.308	99.282	< .001
Event type _(no path change vs. path change)_	−0.003	0.043	5.810	−0.071	.946
Age _(4‐year‐olds vs. 5‐year‐olds)_	−0.056	0.048	92.881	−1.161	.249
Age _(children vs. adults)_	0.198	0.042	92.712	4.703	< .001
Event type _(no path change vs. path change)_: age _(4‐year‐olds vs. 5‐year‐olds)_	−0.007	0.030	983.781	−0.231	.817
Event type _(no path change vs. path change)_: age _(children vs. adults)_	0.026	0.026	983.481	1.033	.302

To interpret the null effect of event type on the latency of button presses, we performed a Bayes factor analysis (Wagenmakers, 2007) using the *brms* package (Bürkner, 2018) in R. The analysis showed that the estimated Bayes factor in favor of the full model including event type as a fixed effect (in addition to age) over the null model that did not include event type as a fixed effect (and only included age) was 0.00262, suggesting evidence in favor of the null model (Jeffreys, 1961; Raftery, 1995).

Visual inspection of Fig. 3 suggested higher dwell times at the beginning and the end of the slideshow as opposed to the moments between them. As an additional check, we explored this pattern statistically. The first and the last slides (Slides 1 and 11) were excluded from this analysis because they are typically associated with unusually high dwell times due to the initial apprehension of the scene. The two middle slides (Slides 5 and 6) were also excluded because they could potentially behave differently depending on event type. We compared button‐press dwell times on the boundary slides (Slides 2 and 10) to those on the non‐boundary slides (the remaining slides after exclusions). We tested the fixed effects of event type, age, and the presence of a boundary on the latency between the button presses. The model also included random intercepts for Subjects and Items. There was a significant effect of the presence of a boundary (*ß* = 0.052, *SE* = 0.008, *t* = 6.727, *p* < .001): participants had longer latencies between button presses for the first and last slides than for non‐boundary slides. These findings suggest an overall pattern in dwell times consistent with attention profiles for event boundaries.

#### 3.2.2 Total duration of fixations

Next, we analyzed the total duration of fixations to each slide. We used the same procedures for transformations and outlier removal as for the button‐press dwell time data. Analyses were performed on log‐transformed total fixation durations and excluding trials that had fixation durations 3 standard deviations above or below the mean (0.6% of all data, children: 0.54%, adults: 0.13%). As in the latency of button presses, fitting a power curve and residualizing was not necessary since the total time participants viewed each slide did not decrease over time.

**Fig. 4:**
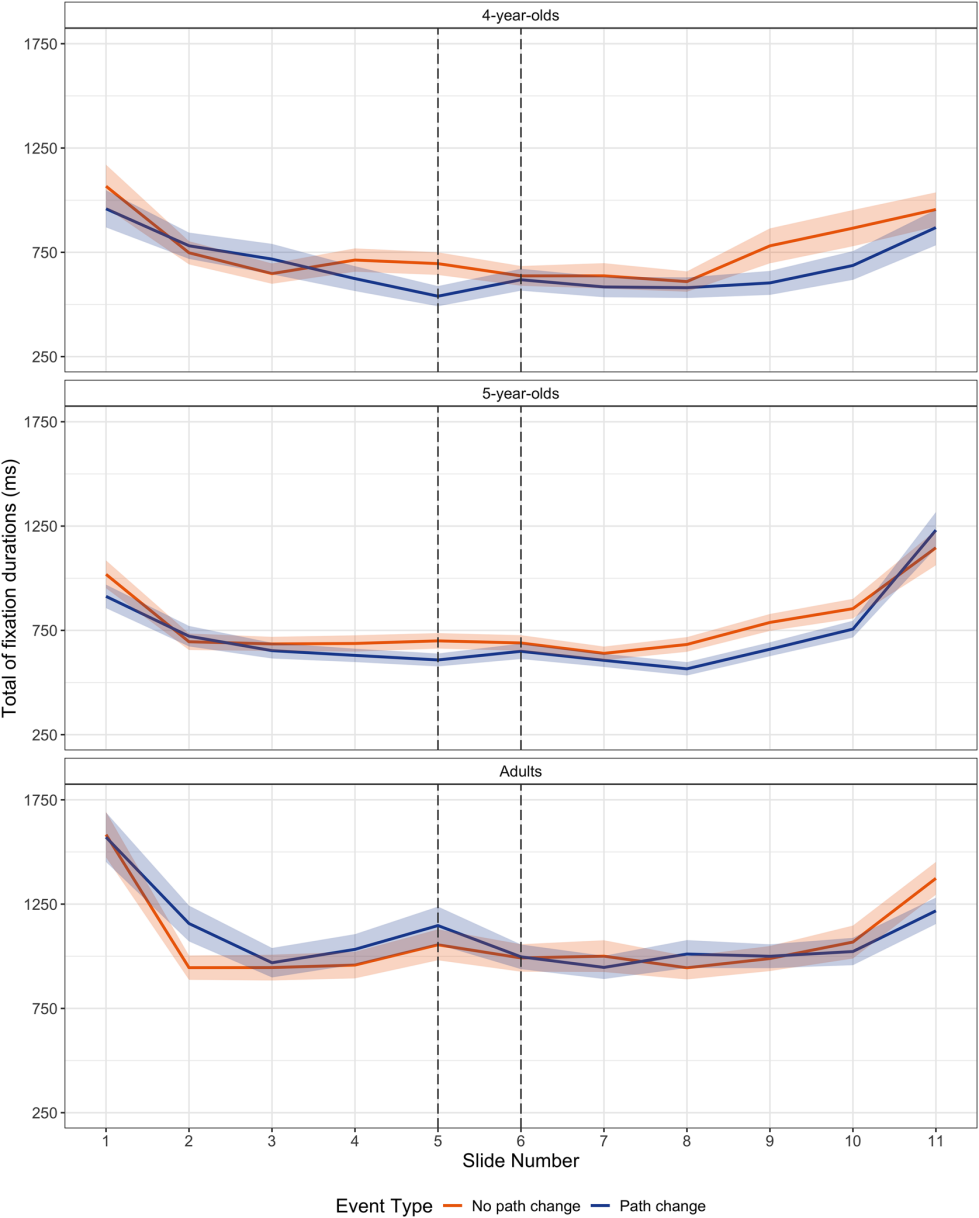
Total fixation durations for each slide across event types and age groups. Shaded areas represent the standard error of participant means.

We tested whether events that involved a change in direction were perceived as having an additional event boundary as indicated by increased total fixation durations for the two middle slides (i.e., slides depicting a path change and the slides leading up to the path change) but not for temporally similar slides that do not depict a path change (Fig. 4). The model tested the fixed effects of event type and age on the total fixation durations to the two middle slides as the dependent measure. Parameter estimates from the *lmer* model are presented in Table 4. The model revealed a significant fixed effect of age at the second contrast level, indicating that adults overall viewed the slides for longer than children did. Importantly, there were no effects or interactions involving event type. This indicated that total fixation durations to slides that depicted a path change were similar to temporally similar slides that did not depict a path change.^3^

**Table 4:** Parameter estimates for the fixed effects from the *lmer* model for total fixation durations

Effect	ß	*SE*	*df*	*t*	*p‐*Value
Intercept	2.856	0.024	18.200	119.128	< .001
Event type _(no path change vs. path change)_	−0.022	0.035	5.791	−0.639	.547
Age _(4‐year‐olds vs. 5‐year‐olds)_	−0.020	0.042	88.412	−0.466	.642
Age _(children vs. adults)_	0.172	0.036	87.575	4.771	< .001
Event type _(no path change vs. path change)_: age _(4‐year‐olds vs. 5‐year‐olds)_	−0.012	0.028	895.075	−0.430	.668
Event type _(no path change vs. path change)_: age _(children vs. adults)_	0.037	0.023	893.897	1.595	.111

To further interpret the null effect of event type on total fixation durations, we performed a Bayes factor analysis. The analysis revealed that the estimated Bayes factor in favor of the full model including event type as a fixed effect (in addition to age) over the null model that did not include event type as a fixed effect (and only included age) was 0.00186, suggesting evidence in favor of the null model (Jeffreys, 1961; Raftery, 1995).

As an additional check, we explored whether the total fixation durations were higher at the beginning and the end of the event, compared to the moments between them as suggested by a visual inspection of Fig. 4. We used the same exclusion and coding criteria as in the analysis of the latency between the button presses to test if the presence of a boundary was associated with longer total fixation durations. The model revealed a significant effect of the presence of a boundary (*ß* = 0.053, *SE* = 0.007, *t* = 7.197, *p* < .001): Participants viewed the slides at the beginning and the end of the event longer than they viewed the slides between them, replicating the pattern for the latency between button presses.

### 3.3 Exploratory analyses

To further evaluate the conclusions drawn from the Dwell Time task, we conducted three sets of exploratory analyses. First, although the stimuli for path‐change events were constructed to reduce predictability of the change in direction, it is possible that path changes might have become more predictable throughout the experiment as direction changes always occurred when the stimuli included an inclined surface. To explore whether the potential increase in the predictability of path changes could have masked differences in the way path‐change and no path‐change events are segmented, we conducted additional analyses focusing on the very first path‐change and no path‐change trials, for which the participants could not have predicted path changes based on stimuli features. Importantly, the analyses did not reveal strong evidence that path‐change versus no path‐change events were segmented differently by children or adults early in the experiment (see the Supporting Information).

Further, given the relatively low number of images in each slideshow in the Dwell Time task, there may not have been enough time for the initially high dwell times to decrease before they could increase again for finer‐grained event boundaries, such as the slides depicting the change in direction as well as anticipatorily for slides leading up to the direction change. This is because dwell times are typically longer at the beginning of the slideshow while participants are processing the scenes. For the same reason, higher dwell times for the beginning and the end of the event may not necessarily be boundary‐specific. To address these issues, we conducted a control experiment with adult participants only by presenting additional scene‐setting slides at the beginning and end of each slideshow and by doubling the number of images in each slideshow. This allowed additional time for participants to process the scene and for initially longer dwell times to decrease before they could possibly increase again. All of the findings from the original experiment were replicated in the control experiment, including higher dwell times for the beginning and the end of the event compared to the moments between them. Findings from the control experiment suggest that the null effect of event type in the original experiment cannot be explained merely by the features of the stimuli or their presentation (see the Supporting Information).

Finally, since there was some variability across participants in the use of multiple verb phrases in the linguistic task, one possibility is that those participants who ended up expressing the presence of a path change using multiple linguistic units had also segmented path‐change and no path‐change events differently in the dwell time task. We addressed this possibility with exploratory analyses of the dwell time data, focusing on the participants who reliably encoded the difference between path‐change and no path‐change events in the linguistic task. The participants who were included in this subgroup had used multiple verb phrases at least 50% more for path‐change events than for no path‐change events. These analyses replicated the findings from the whole sample and found no differences in how path‐change and no path‐change events were segmented (see the Supporting Information). Note that these findings are not surprising given that participants had completed the non‐linguistic dwell time task *before* the linguistic task. In fact, this was done to avoid influence from a task that involves language use to a task that does not. Thus, the non‐linguistic dwell time task measures whether people whose language habitually encodes path segments in multiple linguistic units would also perceive path segments as distinct units even when they are not using language. Another interesting question for future research is whether recent experience with using these linguistic patterns would influence non‐linguistic event segmentation. This can be addressed by having participants completed the non‐linguistic dwell time task *after* the linguistic task.

## 4 Discussion

We experience a continuous world around us, which our minds organize in discrete units in cognition and communication. The way these units are formed in cognition is guided by certain principles (Zacks et al., 2001, 2007; see also Radvansky & Zacks, 2017) as both children and adults can segment events in coarse‐level and fine‐level units (Hard et al., 2011; Kosie & Baldwin, 2021; Meyer et al., 2011; Zheng et al., 2020). Event units in language also express core event components in different levels of granularity (Croft, 1991; Levin & Rapoaport Hovav, 1999) in accordance with the constraints of the structure and typology of a given language (Talmy, 2000). Here, we investigated the relation between event units in language and cognition by asking whether language‐specific event units influence non‐linguistic event segmentation and if such influences change throughout language development.

### 4.1 Linguistic encoding of event units

We take a point of departure from linguistic encoding of motion events in Turkish—a verb‐framed language that typically encodes each path segment in separate linguistic units. We found that Turkish speakers indeed encoded events that had a change in the direction of motion differently than those that had no direction change. Although Turkish‐speaking participants were not more likely to produce descriptions consisting of more than one verb phrase when describing events that had a change in the direction of motion than those without, a closer inspection of the semantic content of the descriptions revealed a reliable difference. Participants were more likely to use more than one path verb to describe events that had a change in direction of motion than those without. This is consistent with our predictions as well as typological patterns documented in previous studies with verb‐framed languages (e.g., Bohnemeyer et al., 2007; Gerwien & von Stutterheim, 2018; Papafragou et al., 2002, 2006; Slobin, 1996). At the same time, for both adults and children, the most common descriptions consisted of single (path) verbs, even for events that had a direction change. Thus, although there was a tendency to map each path segment onto different path verbs, single (path) verb descriptions remained the dominant strategy. This may be explained by the nature of the direction changes, a point we return to below.

Turning to developmental patterns, we also found differences in how frequently children and adults described events using multiple linguistic units. As expected, adults were overall more likely than children to produce descriptions consisting of multiple verb phrases. Further, older children (5‐year‐olds) were more likely than younger children (4‐year‐olds) to describe motion events with more multiple verb phrases, indicating increasing sensitivity to the typological patterns of Turkish in this age range. These developmental differences also persisted for multi‐unit descriptions that encoded the change in the direction of motion with more than one path verb. Specifically, younger group of 4‐year‐olds almost never used multi‐unit descriptions consisting of more than one path verb. Older children did so, though still substantially less frequently than adults did, confirming previously established developmental patterns (Allen et al., 2007; Bunger et al., 2012; Hickmann et al., 2009; Maguire et al., 2010; Özçalışkan & Slobin, 1999; Özyürek et al., 2008). Together, these findings (re‐)establish that linguistic encoding of motion paths provides a good test bed for investigating possible influences of event units in language on non‐linguistic event segmentation and whether such influences might change developmentally during language acquisition.

### 4.2 Event segmentation under minimal linguistic demands

Next, we turn to non‐linguistic segmentation of events in cognition. First, we asked if the way Turkish speakers segmented events would reflect the linguistic units they used for describing them under minimal explicit and linguistic demands. We found that participants segmented events that had a change in the direction of motion similarly to those that did not have a change in direction of motion as indicated by similar visual attention profiles for the two types of events. Notably, this pattern emerged against the backdrop of reliable differences between events that had a direction change and those that did not for explicit boundary judgments in the norming task and event descriptions in the linguistic task. These findings indicate that even though Turkish speakers often used multiple linguistic units expressing motion paths when there was a direction change, they did not appear to segment events into multiple units in cognition based on the same information, at least under conditions that did not encourage the use of language.

At a first glance, these findings seem to be at odds with the findings of a recent cross‐linguistic study (Gerwien & von Stutterheim, 2018) investigating event segmentation in speakers of French and German. In that study, French speakers were more likely than German speakers to indicate an additional event boundary for motion events that had a change in direction of motion, in line with cross‐linguistic differences in event descriptions. However, the differences between these findings and ours may not be surprising given the nature of the non‐linguistic event segmentation tasks across the two studies. The previous study used the Newtson (1973) task that elicited explicit judgments of event boundaries, while we used the Dwell Time paradigm (Hard et al., 2011), which has minimal explicit—including linguistic—demands. As discussed earlier, the explicit and verbal nature of the Newtson task might have encouraged participants to rely on language when judging event boundaries, resulting in discontinuities across speakers of French and German. One aspect of our findings corroborates this explanation. In our norming task, which used similar instructions as the Newtson (1973) task, Turkish speakers were also more likely to indicate the presence of an additional boundary for slides that depicted a change in direction of motion.

By contrast, our findings are consistent with another cross‐linguistic study on event segmentation comparing speakers of Dutch and Avatime (Defina, 2016). Recall that Avatime often uses serial verb constructions to refer to multiple events as a single event. Despite this cross‐linguistic difference, speakers of Avatime and Dutch did not differ in the Dwell Time task. Differences in dwell times only emerged when speakers of Avatime were primed with serial verb constructions. Together, these findings suggest that linguistic influences on event segmentation—if any—seem to be limited to situations in which language is explicitly or implicitly used during non‐linguistic event segmentation.

The present findings also converge with another line of work on motion event expressions in speech and gestures that approach the relation between verbal and non‐verbal event segmentation from a different perspective. This work has shown that when people describe motion events, each co‐speech gesture expresses the semantic information (i.e., path, manner, or both) encoded within a verbal clause in the accompanying speech (Kita & Özyürek, 2003). For example, speakers of Turkish (and other verb‐framed languages) use separate gestures to express manner and path. However, when people are asked to describe motion events only using gestures without accompanying speech, speakers of both Turkish and English conflated path and manner into a single gesture (Özçalışkan, Lucero, & Goldin‐Meadow, 2016, 2018). This contrast in the gestures produced with and without accompanying speech is also reflected in our linguistic and non‐linguistic tasks: Our Turkish‐speaking participants conceptualized multiple motion paths as part of a single event unit even though they distributed the same information into multiple units in language. Overall, these results strongly suggest that Turkish speakers can flexibly shift between different levels of granularity when segmenting events in language and cognition.

### 4.3 Developmental relation between language and event segmentation

As a novel contribution, we also asked whether linguistic influences on event segmentation in cognition would change throughout language development. Here, we entertained two possibilities. One possibility was that children might have segmented events into finer‐level units based on the changes in the direction of motion, despite the fact that adults did not do so. This would indicate that learning to use language‐specific encoding patterns affects the salience of, and the sensitivity to, the cognitive distinctions they encode during acquisition (Bowerman & Levinson, 2001). However, once those language‐specific patterns are fully acquired in adulthood, Turkish speakers would flexibly shift between different levels of granularity depending on whether the use of language is involved (or at least allowed). An alternative possibility was that children, like adults, would segment events differently in language and cognition, indicating a developmentally similar relation between linguistic and non‐linguistic event segmentation. Our findings supported the second possibility. Specifically, children had similar dwell times and visual attention profiles for events that had a change in the direction of motion and those that did not, indicating that they had segmented the two types of events similarly, as did adults. Importantly, children were less likely than adults to use multiple linguistic units in their event descriptions. Despite these differences in language, both adults and children formed single‐event units in cognition and regardless of event type. This highlights that, like cross‐linguistic investigations, developmental comparisons within a single language population can offer valuable insights into the relation between language and cognition by showing whether variations in linguistic encoding patterns are reflected in non‐linguistic behavior.

Notably, there was one developmental difference between children's and adults’ non‐linguistic event segmentation. Adults overall had longer fixation durations than children. This pattern was not specific to events that had a change in the direction of motion or those that did not but generalized across both types of events. These age‐related changes in dwell times and fixation behavior may reflect broader developmental gains in attentional control, such as the ability to disengage from one stimulus and shift attention to another (Diamond, 2013). In fact, these findings cohere with developmental accounts suggesting that age‐related changes in attentional and cognitive abilities are reflected in eye movement patterns (Amso & Scerif, 2015; see also Tanenhaus & Trueswell, 2006; Trueswell, 2008).

Could these developmental differences in visual attention have masked possible linguistic influences on event segmentation reflected in children's dwell times? This seems unlikely for two reasons. First, children—like adults—had longer dwell times at the beginning and end of the event, indicating that they had formed event boundaries. Second, in the linguistic task, children mentioned the changes in the direction of motion, albeit less frequently than adults. This indicates that they could attend to the changes in direction and map these path segments onto distinct linguistic units. Thus, although not at the level of adults, children indeed began shifting between different levels of granularity when segmenting events for linguistic encoding and in non‐linguistic cognition.

Together with our findings from adults, these findings contribute to the broader discussion on the relation between language and cognition. Specifically, our findings go against the position that argues for stable influences of linguistic patterns in non‐linguistic (event) cognition, even under minimal explicit and linguistic demands (Levinson, 2003; Majid et al., 2004). Instead, our findings are consistent with the view that (event) cognition is not influenced by linguistic patterns specific to one's native language, at least in the absence of explicit or implicit language use (Gleitman & Papafragou, 2016; Landau et al., 2010).

### 4.4 Predictability and event segmentation in language and cognition

Although prior work with adults has demonstrated that direction or trajectory changes in an event can increase prediction error, resulting in the formation of event boundaries (Shipley & Macguire, 2008; Zacks, 2004; see also Magliano et al., 2001), this was not the case in our study. Notably, these studies have relied on different kinds of direction changes, such as changes in local geometric features of object paths that do not capture the movement of the object in relation to other objects (Shipley & Macguire, 2008) or changes in velocity or acceleration (Zacks, 2004), which can refer to both collinear and non‐collinear segments. Since our study had a point of departure from cross‐linguistic diversity (cf., Carroll et al., 2012; Gerwien & von Stutterheim, 2018), we focused on a specific kind of direction changes, specifically non‐collinear path segments that are expressed in multiple clauses in some languages and can be expressed in a single clause in other languages because they are inferable (Bohnemeyer et al., 2007). Importantly, some direction changes involving non‐collinear segments simply cannot be expressed as a single clause in any language (Bohnemeyer, 2003; Bohnemeyer et al., 2007). Such direction changes are likely to be less inferable and less predictable and therefore may be more similar in nature to the kinds of direction changes that trigger event boundaries in prior work. Crucially, the way such direction changes are segmented in both language and cognition seems to reflect a cross‐linguistic universal, whereby patterns of linguistic encoding systematically track the cognitive salience of what is unpredictable.

### 4.5 Limitations and future directions

There are some aspects of the present findings that require further attention and open directions for further research. One issue concerns the sensitivity of the dwell times as measured in the current setup to event boundaries. Recall that dwell times were higher at the beginning and the end of the event, compared to the moments between these boundaries, both in the original experiment and in the control experiment with extended slideshows and additional previewing of the scenes. Nevertheless, it remains to be seen if the present paradigm is sensitive to finer‐grained mid‐slideshow event boundaries, which correspond to temporal points other than the beginning and the end of the event. Future work can address this limitation by comparing changes in direction in non‐collinear path segments, which cannot be expressed as a single clause in any language, to the current types of non‐collinear path segments, which show cross‐linguistic variability. Such a comparison would allow for temporally aligning different types of direction changes across items (e.g., mid‐slideshow) and thus would provide stronger evidence to the boundary‐specificity of dwell times.

Another issue, as discussed above, concerns the salience and the predictability of the direction changes. Both adults and children had similar dwell times for slides corresponding to direction changes and temporally similar slides without direction changes, suggesting that our direction changes were not construed as an additional event boundary, possibly because they were predictable. Further, although events that had a direction change were treated differently from those that did not in both linguistic descriptions and explicit boundary judgments, neither the use of multi‐verb descriptions nor the proportion of responses indicating the presence of a boundary was at ceiling. In fact, the majority of the descriptions consisted of single (path) verbs. Future work could systematically manipulate the predictability of direction changes to investigate how they might influence linguistic and non‐linguistic event unit formation.

A related issue concerns the variability in the predictability of the individual path changes in the current stimuli. The stimuli depicting path‐change events were designed to include a brief period of unpredictability, such that when the figure arrives at the base of the inclined surface, different continuations were plausible. Nevertheless, this unpredictability was not identical across the items. In some cases, the unpredictability concerned whether the path would change at all (e.g., continuing straight vs. moving up the incline), whereas in others, it concerned how the path would change among different plausible continuations (e.g., moving into the landmark vs. moving in front of the inclined surface). Further, even when multiple continuations were in principle plausible, the scene might have biased one continuation over the other, resulting in variations in the effective predictability of individual path changes. Such variations might, at least partly, drive the similarities in visual attention profiles for events with and without direction changes. Future work could address this possibility more directly by independently verifying the effective predictability of individual path changes, for instance, by asking participants to anticipate how the event will unfold.

Notably, our stimuli were carefully constructed to control for confounding factors such as changes in camera angle or perspectives, which might otherwise influence dwell times during event segmentation (Meyer et al., 2011; Zheng et al., 2020). This also allowed us to motivate our investigation by distinctions systematically encoded in language. Another interesting avenue for future research is whether segmentation of different classes of events or changes in other core features of event representations might be susceptible to influences of language or other top‐down knowledge, such as goal‐related structure and intentions (Kosie & Baldwin, 2021; Mathis & Papafragou, 2022).

## 5 Conclusion

In conclusion, the present study offers novel insights on the nature of the relation between language and cognition in event segmentation by showing that Turkish‐speaking children and adults segmented events into finer‐level units based on the changes in the direction of motion in language but not in cognition. Together, these results indicate that people flexibly shift between different levels of granularity in event segmentation depending on the linguistic demands of the task.

## Conflict of Interest statement

The authors declare no conflict of interest.

## Supporting information



Supporting Information

## Data Availability

The data that support the findings of this study are available in the Open Science Framework at https://osf.io/ugmyq/.

## Notes

1 Note that the latency of mouse clicks and button presses may slightly differ. However, such differences are unlikely to interfere with our findings as our main predictions are about variations within age groups and response format was always consistent within groups.

2 Two recent studies have examined event segmentation using eye movement measures (Eisenberg & Zacks, 2016; Li et al., 2025). These studies reported that event boundaries were associated with faster eye movements and shorter fixation durations. At first glance, the present approach may appear contradictory to prior work; however, this apparent discrepancy can be attributed to important methodological differences between the stimuli and measures employed. Specifically, both prior studies measured eye movements during continuous viewing of dynamic video stimuli. In contrast, the present study samples static frames at regular intervals from videos and measures the total viewing time of each frame. Consequently, our measure does not rely on the duration of individual fixations per se.

3 A model that included slide number (Slide 5 vs. Slide 6) as an additional fixed effect did not fit the data better and revealed the same findings as the model without the fixed effect of slide number.
