# Supplementary material for: Development of Event Segmentation in Language and Cognition: Evidence From Dwell Times and Eye Movements
Source: Cogn Sci. 2026 Apr 26;50:e70212. doi: 10.1111/cogs.70212 (PMC13110846; doi:10.1111/cogs.70212)
Supplement: Supplementary file 1 — Supporting Information [file COGS-50-e70212-s001.docx]

**Supplementary Material**

**A. Analysis of first trials in the Non-linguistic Dwell Time Task**

The stimuli for path-change events were created in a way that would minimize the change in direction being visually pre-determined and hence predictable. Nevertheless, it is still possible that path-changes might have become more predictable throughout the experiment because direction changes always occurred when the stimuli included an inclined surface (e.g., hill or stairs). In order to explore whether this potential increase in the predictability of path-changes could have masked possible differences in the way path-change and no path-change events are segmented, we conducted additional analyses focusing on the very first path-change and no path-change items that the participants saw.

As in the analysis of all trials, we analyzed the latency between button presses and the total duration of fixations to each slide. Data from both measures were log-transformed. The analyses focused on the slides depicting the path change and the slide leading up the path change as well as temporally similar slides for no path-change events (slides 5 and 6).

***Latency between button presses***

We tested the fixed effects of event type (path-change, no path-change) and age (4-year-olds, 5-year-olds, adults) on button-press dwell times at the item level. We used the same contrast coding scheme as in the analyses of all trials. The model also included random intercepts for Subjects as well as Items. Since item order was reversed for half of the participants, the first item was differed between participants. Parameter estimates from the model are reported in Table S1.

**Table S1. Parameter estimates for the *lmer* model for the latency of the button presses**

| Effect | ß | *SE* | *df* | *t* | *p* value |
| --- | --- | --- | --- | --- | --- |
| Intercept | 2.950 | 0.106 | 2.456 | 27.879 | < .001 |
| Event Type _(No path change vs. Path change)_ | -0.034 | 0.142 | 2.005 | -0.236 | .835 |
| Age _(4-year-olds vs. 5-year-olds)_ | -0.053 | 0.054 | 106.130 | -0.978 | .330 |
| Age _(Children vs. Adults)_ | 0.111 | 0.054 | 105.259 | 2.074 | .040 |
| Event Type _(No path change vs. Path change)_: Age _(4-year-olds vs. 5-year-olds)_ | -0.017 | 0.040 | 235.207 | -0.419 | .676 |
| Event Type _(No path change vs. Path change)_: Age _(Children vs. Adults)_ | 0.101 | 0.040 | 235.421 | 2.552 | .011 |

The model revealed a significant fixed effect of age at the second contrast level, indicating that adults had longer latencies between button presses than children did. Importantly, this effect was also qualified by a significant interaction between age and event type. To interpret this interaction, we conducted pairwise comparisons with corrections for multiple comparisons using *emmans* and *multcomp* packages in *R*. We also plotted the predicted values from the models.

We first followed-up on the interaction between age and event type by examining the fixed effect of event type within each age group. These comparisons revealed that the fixed effect of event type was not statistically significant in any of the age groups (Table S2 and Figure S1).

**Table S2. Parameter estimates for pairwise comparisons for the fixed effect of event type within each age group**

|  |  | *β* | *SE* | *t* | p value |
| --- | --- | --- | --- | --- | --- |
| **4-year-olds** | |  |  |  |  |
|  | No path-change vs. Path-change | 0.034 | 0.142 | 0.236 | .835 |
| **5-year-olds** | |  |  |  |  |
|  | No path-change vs. Path-change | 0.050 | 0.142 | 0.354 | .757 |
| **Adults** | |  |  |  |  |
|  | No path-change vs. Path-change | -0.068 | 0.142 | -0.477 | .681 |

**Figure S1. Predicted values of latency of button presses (event type within age)**

Next, we followed-up on the interaction between age and event type by examining the fixed effect of age within each event type. These comparisons revealed that the age effect between children and adults was statistically significant for both path-change and no path-change events (Table S3). However, the difference between the dwell times of children and adults was larger for path-change events than it was for no path-change events (Figure S2).

**Table S3.** **Parameter estimates for pairwise comparisons for the fixed effect of age within each event type**

|  |  | *β* | *SE* | *t* | p value |
| --- | --- | --- | --- | --- | --- |
| **No path-change** | |  |  |  |  |
|  | 4-yo vs. 5-yo | 0.053 | 0.054 | 0.978 | .592 |
|  | 4-yo vs. Adult | -0.111 | 0.054 | -2.074 | .100 |
|  | 5-yo vs. Adult | -0.164 | 0.053 | -3.105 | .007 |
| **Path change** | |  |  |  |  |
|  | 4-yo vs. 5-yo | 0.070 | 0.054 | 1.278 | .410 |
|  | 4-yo vs. Adult | -0.212 | 0.054 | -3.906 | < .001 |
|  | 5-yo vs. Adult | -0.282 | 0.052 | -5.367 | < .001 |

**Figure S2. Predicted values of latency of button presses (age within event type)**

***Total fixation durations***

We tested the fixed effects of event type (path-change, no path-change) and age (4-year-olds, 5-year-olds, adults) on total fixation durations to each slide at the item level. We used the same contrast coding scheme as in the analyses of all trials. The model also included random intercepts for Subjects as well as Items. Parameter estimates from the model are reported in Table S4.

**Table S4.** **Parameter estimates for the *lmer* model for the total fixation durations**

| Effect | ß | *SE* | *df* | *t* | *p* value |
| --- | --- | --- | --- | --- | --- |
| Intercept | 2.970 | 0.055 | 2.182 | 53.588 | < .001 |
| Event Type _(No path change vs. Path change)_ | -0.014 | 0.107 | 1.903 | -0.128 | .910 |
| Age _(4-year-olds vs. 5-year-olds)_ | -0.004 | 0.040 | 83.262 | -0.114 | .909 |
| Age _(Children vs. Adults)_ | 0.151 | 0.034 | 82.216 | 4.435 | < .001 |
| Event Type _(No path change vs. Path change)_: Age _(4-year-olds vs. 5-year-olds)_ | -0.003 | 0.038 | 239.639 | -0.080 | .936 |
| Event Type _(No path change vs. Path change)_: Age _(Children vs. Adults)_ | 0.089 | 0.032 | 237.346 | 2.789 | .006 |

This model also revealed a significant fixed effect of age at the second contrast level, with adults viewing the slides for longer children, and an interaction between age and event type. We used the same procedures as in the button-press dwell time data to interpret the interaction effect.

We first examined the fixed effect of event type within each age group. These comparisons revealed that the fixed effect of event type was not statistically significant in any of the age groups (Table S5 and Figure S3).

**Table S5.** **Parameter estimates for pairwise comparisons for the fixed effect of event type within each age group**

|  |  | *β* | *SE* | *t* | p value |
| --- | --- | --- | --- | --- | --- |
| **4-year-olds** | |  |  |  |  |
|  | No path-change vs. Path-change | 0.042 | 0.110 | 0.381 | .74 |
| **5-year-olds** | |  |  |  |  |
|  | No path-change vs. Path-change | 0.045 | 0.110 | 0.412 | .72 |
| **Adults** | |  |  |  |  |
|  | No path-change vs. Path-change | -0.046 | 0.109 | -0.419 | .716 |

**Figure S3.** **Predicted values of total fixation durations (event type within age)**

Next, we examined the fixed effect of age within each event type. These comparisons revealed that the age effect between children and adults was statistically significant for both path-change and no path-change events (Table S6). However, the difference between the dwell times of children and adults was larger for path-change events than it was for no path-change events (Figure S4).

**Table S6.** **Parameter estimates for pairwise comparisons for the fixed effect of age within each event type**

|  |  | *β* | *SE* | *t* | p value |
| --- | --- | --- | --- | --- | --- |
| **No path-change** | |  |  |  |  |
|  | 4-yo vs. 5-yo | 0.003 | 0.044 | 0.069 | .997 |
|  | 4-yo vs. Adult | -0.105 | 0.044 | -2.386 | .048 |
|  | 5-yo vs. Adult | -0.108 | 0.043 | -2.491 | .037 |
| **Path change** | |  |  |  |  |
|  | 4-yo vs. 5-yo | 0.006 | 0.045 | 0.137 | .99 |
|  | 4-yo vs. Adult | -0.192 | 0.044 | -4.311 | < .001 |
|  | 5-yo vs. Adult | -0.198 | 0.043 | -4.630 | < .001 |

**Figure S4.** **Predicted values of total fixation durations (age within event type)**

Across both measures, there was no strong evidence that path-change and no path-change events were segmented differently (by children or adults) early in the experiment, before participants could have formed predictions about path changes based on stimuli features. Instead, these effects seem to reflect the overall developmental differences between children and adults. Together, the analysis of the first trials in the dwell time task suggest that the potential increase in the predictability of path changes is less likely to have masked possible differences in how path-change and no path-change events are segmented.

**B. Replication of Non-linguistic Dwell Time Task with extended slideshows**

In the original experiment, the stimuli for the Non-Linguistic Dwell Time Task consisted of 11-image slideshows. Further, the first and last slide in each slideshow depicted a boundary – i.e., the beginning and end of the motion. These features of the stimuli may raise two issues. First, dwell times are usually longer at the beginning of the slideshow while participants are processing the scenes. Thus, longer dwell times for first and last slides may not necessarily be boundary-specific. Second, given the relatively low number of total slides, there may not have been enough time for the initially high dwell times to decrease before they could increase again for finer-grained event boundaries, such as the slides depicting the change in direction as well as anticipatorily for slides leading up to the direction change. To address these issues, we conducted a control experiment with additional scene-setting slides at the beginning and end of each slideshow and by doubling the number of images in each slideshow.

The motion event stimuli were identical to those in the original experiment, with two exceptions. First, the first and last image in each slideshow was repeated three times at the beginning and end of the slideshow. These additional slides served as fillers that set the scene and were excluded from further analysis. Second, the slideshows were created by sampling screenshots from the original videos at a finer temporal resolution. The screenshots were sampled from the videos at 500-milisecond intervals (in contrast to 1-second intervals in the original experiment). As a result, the number of images in each slideshow was doubled, allowing additional time for initially longer dwell times to decrease before they could possibly increase again for middle slides which corresponded to the change in direction for path-change events. The slideshows consisted of a total of 27 slides each, 6 of which were fillers. For path-change events, the middle slide depicted the change in the direction of motion. The middle slide for no path-change events did not depict a change in the direction of motion. Twenty adults participated in the experiment. All were native speakers of English and were recruited from the same participant pool as in the original experiment.

We used the same pre-processing procedures and exclusion criteria as in the original experiment. We excluded 0.3% of the data due to trackloss. We analyzed the latency between button presses and the total duration of fixations to each slide. Data from both measures were log-transformed. The analyses focused on the slides depicting the path change and the slide leading up the path change as well as temporally similar slides for no path-change events (slides 13 and 14).

We tested the fixed effect of event type separately for the latency between button presses and the total duration of fixations to each slide. The models also included random intercepts for Subjects and Items. The models revealed no significant effect of event type on the latency between button presses (*β* = −0.002, *SE* = 0.038, *t* = −0.046, *p* = .965; Figure S5A) or the total fixation durations (β = −0.010, SE = 0.033, t = −0.295, p = .779; Figure S5B). Thus, events that had a change in direction were not perceived as having an additional event boundary than the events that did not have a change in direction.

As a further check, since dwell times can also increase in anticipation of event boundaries, we replicated the analyses above for an earlier window (slides 12 and 13). These models also did not reveal a significant effect of event type on the latency between button presses (*β* = −0.011, *SE* = 0.048, *t* = −0.227, *p* = .827) or the total fixation durations (*β* = −0.025, *SE* = 0.038, *t* = −0.670, *p* = .530).

Finally, we checked whether dwell times would be sensitive to the event boundaries after participants had processed the scene. If so, the slides that depict the beginning and the end of the event should be viewed for longer, compared to the slides that were between event boundaries. To test this, we focused on the slides between the first and last three filler slides (slides 4 to 24). We also excluded the two middles slides (slides 13 and 14). We compared dwell times to boundary slides (beginning: slide 4 and end: slide 24) to those on the non-boundary slides (the remaining slides after exclusions). We tested the fixed effects of event type and the presence of a boundary separately for the latency between button presses and the total duration of fixations to each slide. The models revealed a significant effect of boundary on both latency between button presses (*β* = −0.050, *SE* = 0.015, *t* = −3.363, *p* < .001) and the total fixation durations (*β* = −0.038, *SE* = 0.011, *t* = −3.599, *p* < .001): participants dwelled longer on boundary slides and on non-boundary slides. These findings suggest some boundary-specificity of the dwell times as measured in the current paradigm.

Summarizing, these findings replicate the patterns from the original experiment by showing that adults did not segment path-change and no path-change events differently, with additional time to process the scene and for initially high dwell times to decrease. Therefore, the null effects of event type in the original experiment cannot be explained merely by the features of the stimuli or their presentation.

**Figure S5. Dwell time data in the control experiment with extended slideshows**


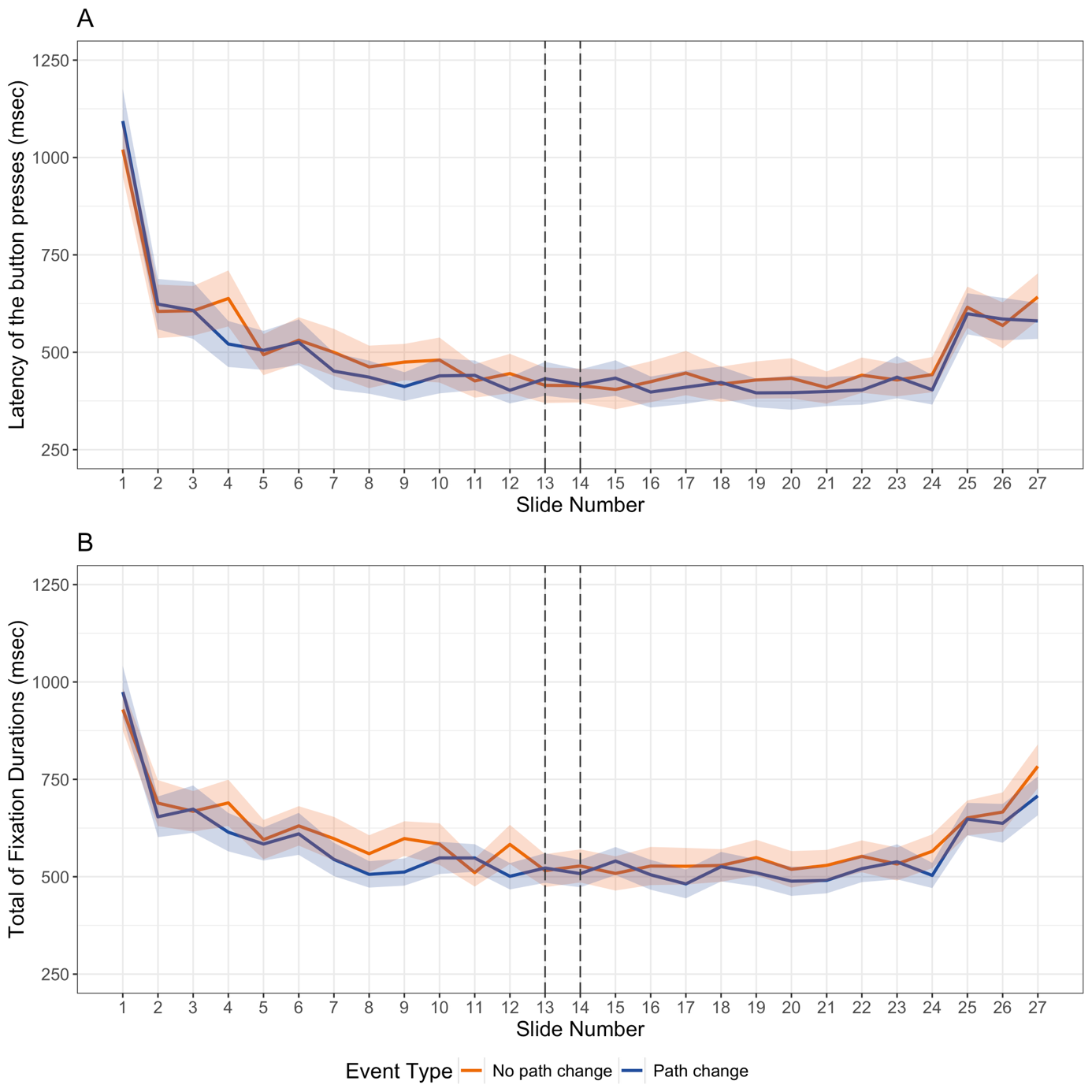


**C. Analysis of the Non-linguistic Dwell Time Task based on the use of multiple verb phrases in the Linguistic task**

In the linguistic task, there was some variability in the use of multiple verb phrases across participants. One might hypothesize that those participants who ended up expressing express the presence of a path change using multiple linguistic units might also be more likely to segment path-change and no path-change events differently in the non-linguistic dwell time task. To address this possibility, we conducted exploratory analyses of the dwell time data, focusing on the participants who reliably encoded the difference between path-change and no path-change events in the linguistic task.

For each participant, we calculated the proportion of descriptions that contained more than one verb phrase separately for path-change and no path-change events. Based on these proportions, we selected those participants who used multiple verb phrases at least 50% more for path-change events than for no path-change events (i.e., proportion of multiple verb phrase for path-change / proportion of multiple verb phrase for no path-change >= 1.50). This classification identified 23 participants (4 four-year-olds, 5 five-year-olds, and 14 adults).

We tested the fixed effects of event type (path-change, no path-change) and age (4-year-olds, 5-year-olds, adults) separately for two the item-level dependent variables: the latency between button presses and the total duration of fixations to each slide. We used the same contrast coding scheme as in the analyses of all trials. The models also included random intercepts for Subjects and Items. Data from both measures were log-transformed. As in the analysis of the whole sample, the analyses focused on the slides depicting the path change and the slide leading up the path change as well as temporally similar slides for no path-change events (slides 5 and 6).

The model predicting the latency between the button presses revealed no significant effect of event type (*β* = −0.005, *SE* = 0.038, *t* = −0.13, *p* = .900) or interaction between event type and age (4-year-olds vs. 5-year-olds: *β* = 0.048, *SE* = 0.077, *t* = 0.63, *p* = .532; children vs. adults: *β* = 0.029, *SE* = 0.048, *t* = 0.62, *p* = .538). As in the analysis of the whole sample, there was a significant fixed effect of age at the second contrast level (*β* = 0.246, *SE* = 0.102, *t* = 2.42, *p* = .025): adults had longer latencies between button presses than children did.

The model predicting total fixation durations also did not reveal a significant effect of event type (*β* = −0.041, *SE* = 0.028, *t* = −1.44, *p* = .192) or interaction between event type and age (4-year-olds vs. 5-year-olds: *β* = 0.098, *SE* = 0.073, *t* = 1.34, *p* = .182; children vs. adults: *β* = 0.036, *SE* = 0.045, *t* = 0.80, *p* = .427). As in the analysis of the whole sample, there was a significant fixed effect of age at the second contrast level (*β* = 0.183, *SE* = 0.079, *t* = 2.31, *p* = .032): adults viewed the slides for longer than children did.

Finally, given the relatively small number of children meeting the criterion for inclusion in this subgroup analyses, we conducted a follow-up analysis restricted to adult participants. We tested the fixed effect of event type separately for the latency between button presses and the total duration of fixations to each slide. The models revealed no significant effect of event type on the latency between button presses (*β* = 0.014, *SE* = 0.041, *t* = 0.35, *p* = .745) or the total fixation durations (*β* = −0.018, *SE* = 0.033, *t* = −0.53, *p* = .626).

Overall, these findings replicate the findings from the analyses of the whole sample. That is, even the subgroup of participants who ended up expressing the presence of a change in direction later in the linguistic task, did not have previously segmented events that had a direction change differently from the events that did not have a direction change.

Note that this finding is not surprising given that participants had completed the non-linguistic dwell time task *before* the linguistic task. In fact, this was done to avoid influence from a task that involves language use to a task that does not. Thus, the non-linguistic dwell time task measures whether people whose language habitually encodes path segments in multiple linguistic units would also perceive path segments as distinct units even when they are not using language. Another interesting question is whether recent experience with using these linguistic patterns would influence non-linguistic event segmentation. Future work can address this by having participants completed the non-linguistic dwell time task *after* the linguistic task.
